# The dataset for validation of factors affecting teachers’ decision to integrate character values into curriculum

**DOI:** 10.1016/j.dib.2022.108404

**Published:** 2022-06-20

**Authors:** Muzakkir, Zaharah Hussin, Rafiza Abdul Razak, Ahmad Munir

**Affiliations:** a Department of Curriculum and Instructional Technology, Faculty of Education, University of Malaya, 50603, Kuala Lumpur, Malaysia; b Department of Educational Foundations & Humanities, Faculty of Education, University of Malaya, 50603 Kuala Lumpur, Malaysia; c LPDP Indonesia, Gedung Dandyaksa Cikini Raya No. 91A-D, Menteng, Jakarta Pusat, Indonesia; d Universitas Negeri Makassar, Jalan A. P. Pettarani, Tidung, Kecamatan Rappocini, Kota Makassar, Sulawesi Selatan, Indonesia

**Keywords:** Character education, Curriculum, Teachers’ beliefs, Pedagocical content knowledge, Character values integration, Teachers’ decision

## Abstract

The objective of this dataset is to examine the effect of factors of pedagogical content knowledge (PCK) and teachers’ beliefs (TB) on teachers' decisions (TD) to select character values to integrate into the curriculum in primary school in Indonesia. The data propose that PCK factors and teachers' beliefs (TB) factors significantly influence TD. PCK factors consist of content knowledge (CK), pedagogical knowledge (PK), and pedagogical content knowledge (PCK). While, TB factors consist of Attitude (ATT), Subjective norm (SN), and perceived behavioural control (PBC). The survey approach obtained 50 responses from one public school and two private schools in Indonesia. After adapting the survey instrument, face and content validity were conducted. Further, to examine the validity and reliability of the measurement model, a Partial Least Squares Structural Equation Model (PLS-SEM) was applied. For this purpose, the statistical process presents the load of the reflection indicator, the reliability of internal consistency, and the validity of convergence and discrimination. The dataset consists of demographic information, PCK, beliefs on character values integration, and teachers' decision to select character values to integrate into the curriculum. The dataset is beneficial to curriculum developers, school principals, and teachers for measuring factors affecting teachers' decisions to integrate character values into the curriculum.

**Specifications Table**

**Table:** 

Subject	Education
Specific subject area	Character education, curriculum
Type of data	TableFigure
How the data were acquired	Face and content validity, survey and PLS-SEM.
Data format	Raw, Analyzed, Filtered
Description of data collection	Demographic information, PCK, beliefs on character values integration, teachers’ decision to integrate character values into curriculum
Data source location	Data gathered from three schools in Balikpapan, Batam, and Depok, Indonesia
Data accessibility	On a public repository name: Mendeley Data identification number: 10.17632/dd7hnsk4xf.1 Direct URL to the data:https://data.mendeley.com/datasets/dd7hnsk4xf/3

**Value of the Data**

• Valid and reliable dataset are important to support studies regarding character values integration
• in education.
• Practically, the data are beneficial for curriculum development centers, teachers, and school leaders to integrate proper character values into curriculum.
• The dataset could be adopted, adapted, or extended for future researchers interested in conducting research with similar topics.

## 1 Data Description

**Fig. 1:**
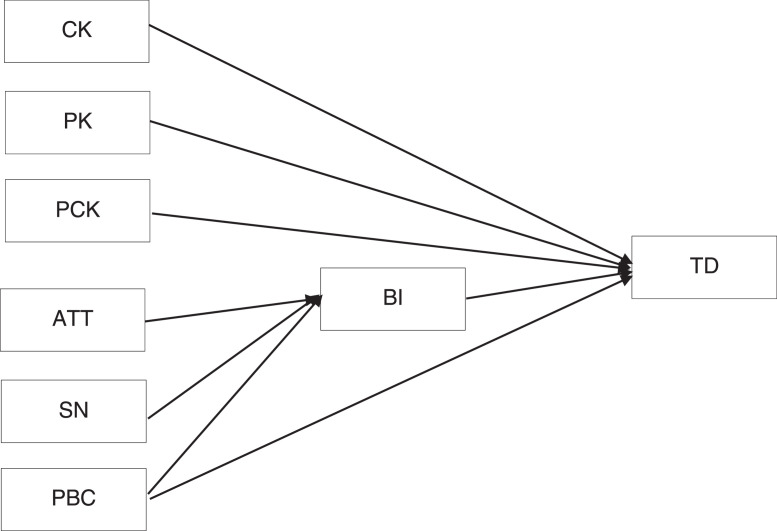
Proposed model.

**Fig. 2:**
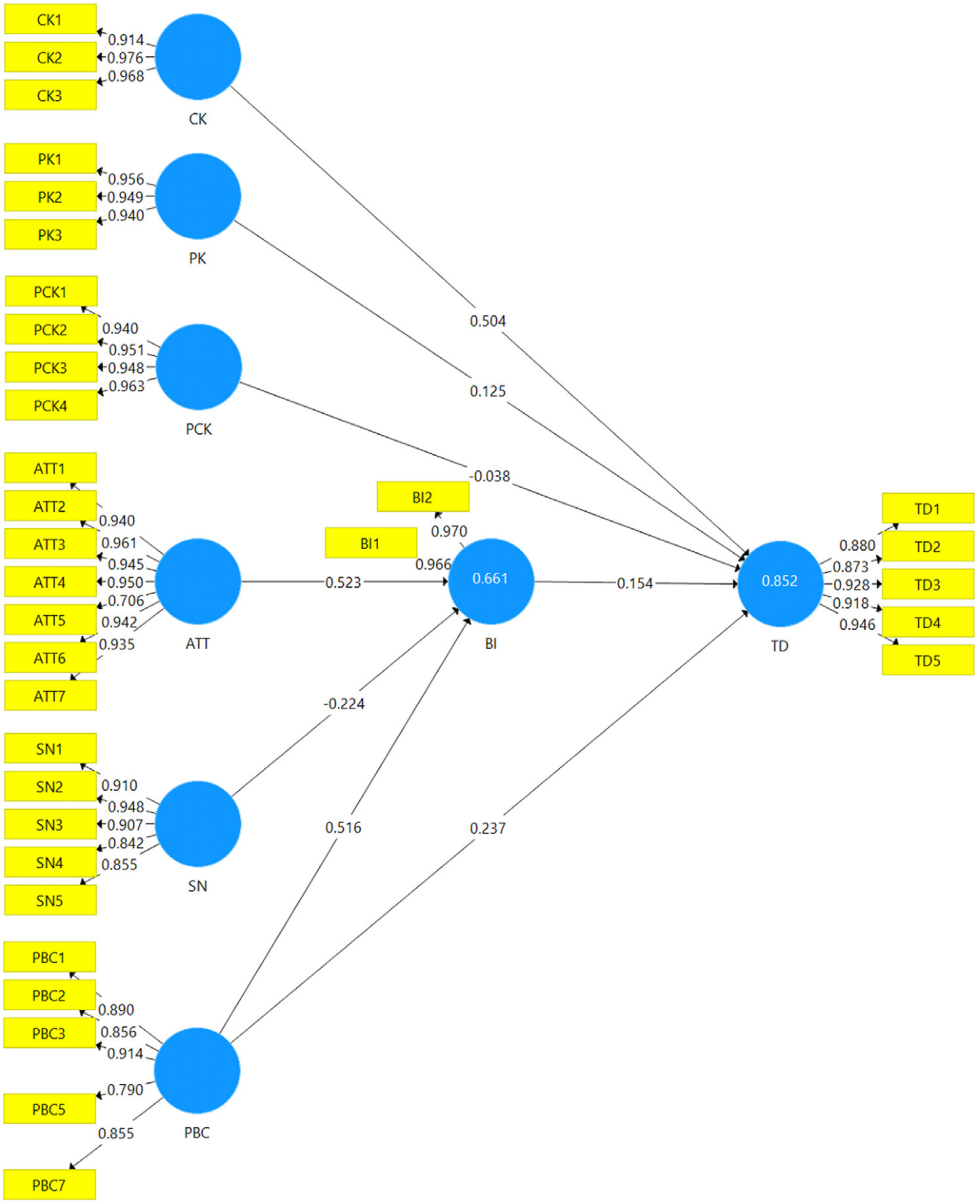
Measurement model.

This dataset proposes that pedagogical content knowledge (PCK) factors and teachers' beliefs factors significantly influence teachers' decision (TD) to integrate character values into the curriculum. PCK factors include content knowledge (CK), pedagogical knowledge (PK), and pedagogical content knowledge. Teachers' beliefs factors include attitude (ATT), subjective norms (SN) and perceived behavioural control (PBC). CK is a professional competency to master widely and deeply learning content, including mastery of curriculum content, taught subject matter at school. PK is described as teachers' ability to meaningfully deliver the lesson integrated with character values to students in the classroom. Meanwhile, a competency to combine two knowledge, CK and PK, to become part of the teaching process is defined as PCK. ATT is defined as teachers' feelings or mental state about the decision to select character values to integrate into the curriculum. At the same time, SN is described as teachers' beliefs that other people support them in determining character values to incorporate into the curriculum. PBC is defined by teachers' views regarding the availability of resources to assess character values to integrate into the curriculum. BI refers to teachers' intention to select character values to incorporate into the curriculum. Meanwhile, TD relates to teachers' decision to integrate character values into the curriculum. The dataset includes two sections, namely demographic information and the main survey. The demographic questions include age, teaching experience, and school status (Table 1). While the main survey has six exogenous and two endogenous constructs (Fig. 1). Six exogenous are three constructs included in PCK measured from 1 = strongly disagree to 5 = strongly agree are CK (3 items), PK (3 items), and PCK (4 items), adapted from previous academic research [1,2], and three constructs included in teachers' beliefs which are ATT (7 items), SN (5 items), and PBC (7 items)). The last constructs were two endogenous constructs, namely BI (2 items) and TD (5 items) [3], [4], [5]. Table 2 exhibits the Mean, Standard Deviation, Skewness and Kurtosis of the data. Table 3 provides the information on the three assessments of the measurement model (reflective indicator loadings, internal consistency reliability, and convergent validity). Tables 4 and 5 show the discriminant validity by evaluating the Fornell-Larcker criterion and cross-loading. Fig. 2 exhibits the measurement model of the dataset. The raw dataset and instrument are accessible on https://data.mendeley.com/datasets/dd7hnsk4xf/3.

**Table 1:** Demographic Information (n. 50).

Demographic	*n*	%
*Age*		
20 - 30 years	20	40.0
31 – 40 years	14	28.0
41 – 50 years	12	24.0
> 50 years	4	8.0

*Teaching Experience*		
0 – 5 years	21	42.0
6 – 10 years	15	30.0
11 – 15 years	11	22.0
16 – 20 years	1	2.0
> 20 years	2	4.0

*School Status*		
Public school	14	28.0
Private school	36	72.0

**Table 2:** Mean, SD, skewness, and kurtosis.

			Skewness		Kurtosis	
	M	SE		Std. Error		Std. Error
CK1	2.3400	.13883	.873	.337	.750	.662
CK2	2.2800	.16670	1.216	.337	.759	.662
CK3	2.1600	.15743	1.150	.337	.846	.662
PK1	2.2800	.17389	1.014	.337	.234	.662
PK2	2.3000	.14070	1.036	.337	.876	.662
PK3	2.3000	.14070	.907	.337	.763	.662
PK4	2.3800	.15089	.957	.337	.484	.662
PCK1	2.2200	.15982	1.048	.337	.485	.662
PCK2	2.3200	.16522	.935	.337	.232	.662
PCK3	2.3000	.15186	.907	.337	.186	.662
ATT1	2.0800	.18034	1.259	.337	.590	.662
ATT2	2.1800	.17541	1.248	.337	.605	.662
ATT3	2.2600	.17335	1.070	.337	.348	.662
ATT4	2.1800	.18225	1.140	.337	.190	.662
ATT5	2.5800	.13729	.253	.337	-.417	.662
ATT6	2.4800	.17436	.967	.337	.130	.662
ATT7	2.4000	.14846	.992	.337	.577	.662
SN1	2.5200	.15969	.701	.337	-.069	.662
SN2	2.4600	.14627	.688	.337	.442	.662
SN3	2.6200	.12085	-.186	.337	-.483	.662
SN4	2.7200	.13714	.182	.337	-.043	.662
SN5	2.7000	.14639	-.046	.337	-.272	.662
PBC1	2.4800	.18124	.773	.337	-.430	.662
PBC2	2.5800	.14311	.573	.337	-.218	.662
PBC3	2.3000	.15972	.877	.337	.176	.662
PBC5	2.5200	.14912	.544	.337	.121	.662
PBC7	2.4400	.15162	.680	.337	-.236	.662
BI1	2.3200	.12269	.682	.337	.879	.662
BI2	2.4200	.13429	.686	.337	.050	.662
TD1	2.4400	.13750	.800	.337	.510	.662
TD2	2.3200	.13833	.667	.337	.061	.662
TD3	2.2600	.12392	.593	.337	-.141	.662
TD4	2.3000	.11157	.701	.337	.301	.662
TD5	2.2800	.11443	.640	.337	.183	.662

**Table 3:** Reflective indikator loadings, internal consistency, composite reliability, and convergent validity.

		Load	α	CR	(AVE)
ATT	ATT1	.940	.966	.973	.838
	ATT2	.961			
	ATT3	.945			
	ATT4	.950			
	ATT5	.760			
	ATT6	.942			
	ATT7	.935			

BI	BI1	.966	.933	.967	.937
	BI2	.970			

CK	CK1	.914	.949	.967	.908
	CK2	.976			
	CK3	.968			

PBC	PBC1	.890	.913	.935	.743
	PBC2	.856			
	PBC3	.914			
	PBC5	.790			
	PBC7	.855			

PCK	PCK1	.931	.973	.979	.903
	PCK2	.950			
	PCK3	.944			
	PCK4	.966			
	PCK5	.958			

PK	PK1	.956	.944	.964	.899
	PK2	.949			
	PK3	.940			

SN	SN1	.910	.939	.952	.798
	SN2	.948			
	SN3	.907			
	SN4	.842			
	SN5	.855			

TD	TD1	.880	.948	.960	.872
	TD2	.873			
	TD3	.928			
	TD4	.918			
	TD5	.946

**Table 4:** Fornell-larcker criterion.

	ATT	BI	CK	PBC	PCK	PK	SN	TD
ATT	.915							
BI	.779	.968						
CK	.904	.777	.953					
PBC	.851	.770	.837	.862				
PCK	.892	.744	.926	.804	.951			
PK	.854	.730	.933	.800	.941	.948		
SN	.815	.641	.765	.850	.754	.710	.893	
TD	.866	.790	.903	.846	.851	.861	.707	.910

**Table 5:** Cross loading.

	ATT	BI	CK	PBC	PCK	PK	SN	TD
ATT1	**.940**	.737	.810	.802	.761	.721	.739	.814
ATT2	**.961**	.733	.833	.813	.784	.773	.744	.817
ATT3	**.945**	.732	.853	.765	.830	.773	.752	.811
ATT4	**.950**	.759	.863	.838	.832	.800	.749	.839
ATT5	**.706**	.568	.722	.598	.799	.781	.620	.643
ATT6	**.942**	.736	.839	.806	.818	.801	.815	.810
ATT7	**.935**	.709	.868	.803	.864	.842	.793	.798
BI1	.721	**.966**	.712	.733	.687	.685	.618	.734
BI2	.785	**.970**	.789	.757	.743	.727	.622	.794
CK1	.795	.690	**.914**	.714	.852	.837	.708	.749
CK2	.856	.754	**.976**	.815	.887	.915	.704	.910
CK3	.928	.772	**.968**	.853	.912	.911	.776	.908
PBC1	.796	.636	.726	**.890**	.690	.649	.813	.737
PBC2	.700	.625	.668	**.856**	.641	.618	.762	.684
PBC3	.748	.646	.743	**.914**	.706	.714	.795	.729
PBC5	.650	.680	.649	**.790**	.638	.660	.624	.674
PBC7	.763	.722	.803	**.855**	.779	.789	.673	.807
PCK1	.843	.695	.852	.750	**.931**	.885	.682	.828
PCK2	.856	.727	.881	.770	**.950**	.900	.714	.780
PCK3	.852	.716	.914	.788	**.944**	.912	.749	.819
PCK4	.842	.690	.872	.750	**.966**	.881	.723	.806
PCK5	.810	.686	.885	.766	**.958**	.919	.708	.805
PK1	.804	.723	.914	.782	.925	**.956**	.694	.863
PK2	.825	.710	.882	.765	.868	**.949**	.664	.813
PK3	.801	.640	.856	.727	.899	**.940**	.658	.769
SN1	.898	.748	.832	.796	.808	.776	**.910**	.790
SN2	.790	.639	.708	.794	.675	.651	**.948**	.662
SN3	.667	.530	.633	.733	.641	.574	**.907**	.574
SN4	.576	.375	.579	.734	.614	.579	**.842**	.511
SN5	.580	.416	.572	.739	.555	.509	**.855**	.515
TD1	.785	.760	.826	.770	.745	.725	.713	**.880**
TD2	.745	.713	.745	.766	.666	.666	.575	**.873**
TD3	.799	.720	.864	.772	.828	.857	.635	**.928**
TD4	.756	.675	.807	.754	.798	.827	.566	**.918**
TD5	.848	.726	.857	.786	.823	.830	.719	**.946**

## 2 Experimental Design, Materials and Methods

We applied 2-phase procedures in this study for scale development. Phase 1 is the adaptation and translation of the research instrument. We adapted the research instrument referring to previous literature sources, followed by the translation of the scale. The scale was translated from English to Indonesian and Indonesian to English using a reverse translation method involving two experts. In phase 2, face and content validity were conducted with two discussion sessions. The first session was a discussion with three users to ensure that the instrument was easy to understand by the sample respondents. The next session was a discussion with three experts to evaluate the scale for the appropriateness of context and setting. We then did an online survey based on google form to collect the data from March to April 2022 through simple random sampling. We randomly selected a subset of participants from the population who are primary school teachers in three Indonesian provinces, Balikpapan, Batam, and Depok. After receiving all responses, we converted the data into Microsoft Excel. Firstly, we assessed the normality by calculating Skewness and Kurtosis in SPSS 25, in which the values should be between -2 to + 2 [6]. All Skewness and Kurtosis values are in the range of the threshold; Skewness (SN3, -186 to ATT1, 1.259) and Kurtosis (BI1, 0.879 to ATT5, -417) (Table 1). We then reported the four assessments of the measurement model (reflective indicator loadings, internal consistency reliability, discriminant and convergent validity) using the approach of PLS-SEM in Smart PLS 3.2. The loading of the reflective indicator should be .708 or higher. Table 2 performs all loading values that fulfil the threshold (.760- .968). We dropped two items (PBC4 and PBC6) due to their low loading values. Cronbach's alpha and Composite Reliability (CR) of greater than .700 should be applied for the internal consistency [6,7]. The Cronbach's alpha values of this dataset range from .913 to .973; similarly, the CR values are between .935 and .979. The validity of convergent was reported through Average Variance Extracted (AVE); the value of .500 or higher is recommended [8]. The AVE values range from .743 to .937 (Table 2). The discriminant validity was evaluated by using the Fornell-Larcker and cross-loading. The AVE values of a construct should be less than the shared variance of the Fornell-Larcker's other construct. The data showed that the values of every construct are less than its’ shared variance (Table 4). The discriminant validity is reported when loading on a construct is greater than those of other constructs; cross-loading values. The values for all indicators (bold) in each construct exceeded all their cross-loadings (Table 5). The model consists of eight constructs with 35 indicators (Fig. 2).

## Ethics Statements

Informed consent was obtained for the data collection and the participation was voluntary. The survey was anonymous that did not include any personal information of the participants.

## CRediT authorship contribution statement

**:** Conceptualization, Methodology, Software, Data curation, Investigation. **Zaharah Hussin:** Conceptualization, Supervision. **Rafiza Abdul Razak:** Conceptualization, Supervision. **Ahmad Munir:** Software, Validation, Visualization, Writing – original draft.

## Declaration of Competing Interest

The authors declare that they have no known competing financial interests or personal relationships that could have appeared to influence the work reported in this paper.
